# Harnessing bioengineered myeloid progenitors for precision immunotherapies

**DOI:** 10.1038/s41536-023-00343-x

**Published:** 2023-12-12

**Authors:** Willem Buys, Elias T. Zambidis

**Affiliations:** Institute for Cell Engineering, and Division of Pediatric Oncology, Sidney Kimmel Comprehensive Cancer Center, The Johns Hopkins University School of Medicine, Baltimore, MD USA

**Keywords:** Stem-cell research, Stem-cell biotechnology

## Abstract

Granulocytes and macrophages are the frontline defenders of the innate immune system. These myeloid cells play a crucial role in not only eliminating pathogens and tumor cells, but also regulating adaptive immune responses. In neonatal sepsis and post-chemotherapy agranulocytosis, the absence of these cells leaves the host highly vulnerable to infections. Beyond replacement to prevent or control neutropenic sepsis, engineered myeloid cells may offer distinct opportunities for cell therapies. For example, the mobility and specific homing capacities of neutrophils to sites of inflammation could be exploited to deliver biocidal agents, or anti-inflammatory healing signals during sepsis, autoimmunity, and organ transplantation. Additionally, myeloid cells can be engineered to express chimeric antigen receptors (CAR), carry chemotherapeutics, or enhance lymphoid tumor killing. However, traditional methods of cell isolation are incapable of providing sufficient cell numbers of these short-lived cells; their propensity for premature activation further complicates their cell engineering. Here, we review current and future biotherapeutic innovations that employ engineered multipotent myeloid progenitors derived from either self-renewing human induced pluripotent stem cells (hiPSC) or primary CD34^+^ hematopoietic stem-progenitors. We provide a roadmap for solving the challenges of sourcing, cost, and production of engineered myeloid cell therapies.

The hematopoietic system arises from self-renewing hematopoietic stem cells (HSC), that possess the ability to reconstitute all blood lineages. While early fate decisions are incompletely understood, hematopoiesis generally branches into multipotent lymphoid and myeloid progenitors. A common lymphoid progenitor generates Natural Killer-, B-, and T- cells. The common myeloid progenitor, in turn, generates megakaryocyte-erythroid-, mast cell-, and granulocyte-macrophage progenitors, which produce erythrocytes and platelets, mast cells, and granulocytes and macrophages. The macrophage family is comprised of myeloid dendritic cells (DC) and monocytes, which can mature to tissue-resident macrophages in the periphery, while granulocytes are comprised of neutrophils, eosinophils, and basophils. Neutrophil granulocytes are the most abundant type of immune cell and specialize in pathogen recognition and killing. They can effectively home to sites of inflammation beyond physiological barriers (e.g., the blood-brain barrier), release biocides, phagocytose pathogens, and regulate other immune cells, including lymphocytes. However, granulocytes have limited lifespans, and their numbers rapidly decline following bone-marrow damage (e.g., during neonatal sepsis, radiotherapy, and chemotherapy). In contrast, cells of the macrophage family with similar functionalities are longer-lived, potentially more prolific, and focus less on pathogen killing and more on phagocytosis, mediator production, and antigen presentation.

Although the therapeutic application of myeloid cells is limited by cell availability, recent advances with human induced pluripotent stem cells (hiPSC) and ex vivo amplification and differentiation of primary hematopoietic stem-progenitor cells now enable the amplification of transiently engrafting myeloid progenitors from primary HSC or from hiPSC and have renewed an interest in engineered granulocyte and macrophage cell therapies. Myeloid progenitors may simplify cell engineering strategies by providing an “off-the-shelf” solution with manyfold lesser cell numbers required for transfusion than terminally differentiated granulocytes or macrophages, and with an extended duration of effect, following in vivo amplification. Here, we highlight the potential utility of myeloid immune therapies, which spans beyond treatment of neutropenic sepsis, and includes drug delivery, and pro-inflammatory regulation to enhance tumor or pathogen killing^1,2^, or anti-inflammatory regulation^3,4^, such as in organ transplant rejection. We discuss and contrast the merits of engineered myeloid progenitor cell-therapies versus repeated transfusions of end-differentiated myeloid cells in various applications. We prioritize specific bioengineering strategies for improving myeloid immune therapies, including via myeloid-based chimeric antigen receptors (CAR)^5^, targeted regulation of tumor immune-microenvironments^6,7^, optimization of large-scale cell manufacturing^8,9^, and drug delivery approaches with either whole myeloid cells or myeloid cell membrane-coated nanoparticles^10^.

## Therapy of neutropenic sepsis

Severe neutropenia critically impairs primary inflammation and host defenses^11^. Despite prophylactic administration of antibiotics and granulocyte-colony stimulating factor^12^, life-threatening sepsis from severe neutropenia remains a major driver of morbidity and mortality during neonatal sepsis, or following chemotherapy or prep conditioning for bone-marrow transplantation (BMT) (Table 1), with a mortality rate of ≈7%^13^. Experimental approaches to support endogenous production, extend the lifespan, or enhance the inflammatory activities of myeloid effector cells^14^ to overcome infection susceptibility are ultimately limited by availability of endogenous progenitors, and may only be amenable to mild cases of chemotherapy-associated neutropenia.

**Table 1:** Estimated need for granulocyte replacement therapies.

Cause of Neutropenia	Absolute incidence (USA)	Rates of severe neutropenia	Neutropenia	
			Absolute incidence (USA)	Incidence rate per million
HSC transplantation	22,000	100%	22,000	67
Chemotherapy	≥500,000^a^	11%	55,000	167
Non-chemotherapy drug neutropenia	800–5100	-	≤800–5100	9
Neonatal sepsis	3500–16,000	Unclear^**b**^	≤3500–16,000	30
Sum			55,000–95,900^c^	273

Severe neutropenia is defined as <500 neutrophils/µl blood, including grade 3/4 neutropenia (“severe”/“life-threatening”; <1000/µl) per Common Terminology Criteria for Adverse Events^78^.

^a^No definitive data available. Estimates are under the assumption that all persons who die from cancer (≈150/100,000^79^) will have received at least one course of chemotherapy. This is consistent with data available^13^. As this estimate is not accounting for cancer survivors and multiple courses of chemotherapy, the true number is likely higher.

^b^Neutropenia is cited as a common complication of neonatal sepsis, but accurate frequency data is not available.

^c^Also accounts for possible intersectionality (HSC and chemotherapy).

A natural solution is to bridge this hiatus by transfusing donor granulocytes until normal bone marrow function can return. While a plethora of studies have reported improved pathogen clearance and trends towards better survival following granulocyte transfusions^15,16^, there is a lack of definitive data demonstrating therapeutic benefit^17^. Large studies are hindered by the complex logistics of generating enough of these short-lived cells^15,16^. Furthermore, terminally differentiated granulocytes lose both functionality and viability within hours-to-days and cannot be effectively cryopreserved^16^. Hence, even using the most effective method of granulocyte production via donor-priming and apheresis, the biggest clinical trial published to date failed to consistently generate >10^10^ cells per transfusion; this limitation was reported as a major factor for not observing a significant survival benefit^18^. Although larger numbers of granulocytic cells for transfusion could be generated in vitro from primary CD34^+^ cells or hiPSC, the need for large batch productions would further drive associated costs and delay provision. Even if accepting the cost of weeks of in vitro production^8,9^, or donor-sided unwanted effects of priming and apheresis^16^, the adequate provision of terminally differentiated granulocytes for transfusion is surprisingly ineffective compared to endogenous natural production. Accordingly, the practice of transfusing terminally differentiated granulocytes to treat or prevent neutropenic infections currently remains limited in its practice.

**Fig. 1:**
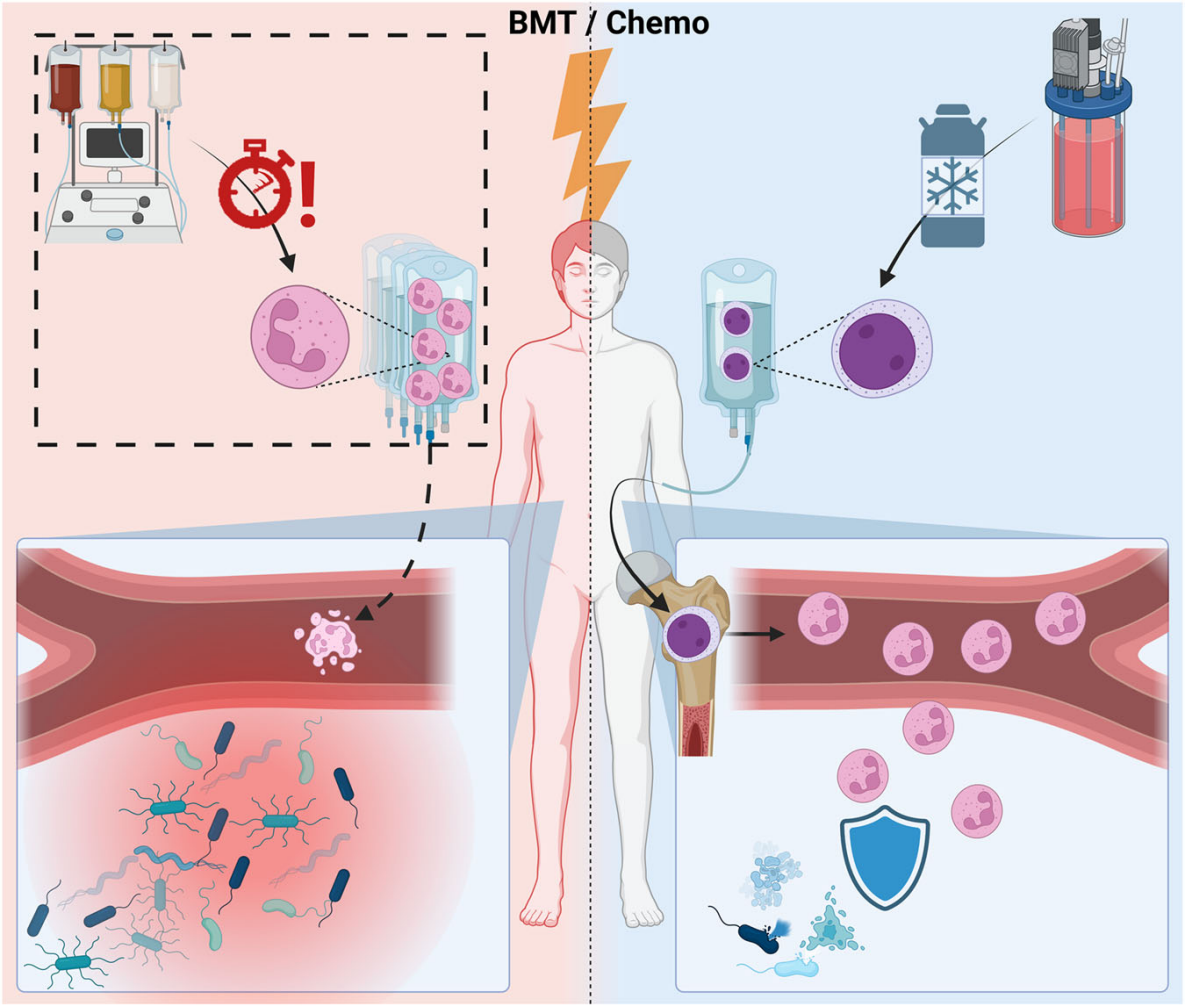
In vivo amplification of myeloid progenitors for protection from neutropenic sepsis. Despite growth factor prophylaxis, severe neutropenia following chemotherapy, or a bone-marrow transplantation prep, leaves the host highly susceptible to infection. Neutrophil transfusion (left panel) requires prohibitive cell numbers that cannot regularly be provided using available methods. Isolation is highly time sensitive, and the product cannot be stored or frozen effectively. In contrast, myeloid progenitors (right panel) differentiated in vitro from primary CD34^+^ stem-progenitor cells or hiPSCs could be cryopreserved and engrafted for a short-term production of effector cells, thus potentially reducing infectious complications.

One possible solution may be to augment the production of terminally differentiated granulocytes by transfusing more prolific, longer-lived, and transiently engrafting myeloid progenitors into neutropenic hosts (Fig. 1). For example, in a Phase 2 clinical study, Desai et al demonstrated reduced infections and shorter hospital stays following a single infusion of myeloid progenitors incapable of long-term engraftment derived from GCSF-mobilized CD34^+^ peripheral-blood stem-progenitor cells (PBSC)^19^. The temporary engraftment and in vivo expansion and differentiation of progenitors allowed for a single administration, thus significantly lowering the cell numbers required for clinical efficacy. Additionally, myeloid progenitors were freeze-thaw tolerant enabling a viable “off-the-shelf” provision. This technique earned the developing company a Regenerative Medicine Advanced Therapy Designation by the U.S. Food and Drug Administration, and a Phase 3 study is currently in progress^20^. Consistent with earlier observations that unmatched granulocyte transfusions rarely caused complications^21^, Desai et al did not report significant adverse effects of HLA-unmatched progenitor transfusion (albeit without reporting the frequency of alloimmunization)^19^.

Both murine and human studies have reported a delay of ≈5–6 days from progenitor cell infusion to effect onset^6,19^. As neutropenia can be predicted with a reasonable probability in chemotherapy and BMT settings, this technique is promising for ameliorating the risks of expectant iatrogenic neutropenia; the most common cause of neutropenia (Table 1). Moreover, HLA-mismatching could serve as an additional safety mechanism that prevents permanent hematopoietic chimerism in cases of contamination with long-term engraftment-competent HSC within PBSC populations^22^.

## Immune regulation and tolerance

Although myeloid immune cells serve primarily pro-inflammatory functions, they also perform regulatory and anti-inflammatory roles^23^. Immune-regulatory subtypes within various myeloid populations include myeloid-derived suppressor cells (MDSC), M2 polarized macrophages, tolerogenic DC, and possibly low-density neutrophils. Within the larger myeloid family, even erythrocyte progenitors have been inferred in immune regulation and tolerance and considered as cell therapeutics (rev^24^.).

In mice, transfer of MDSC improved airway resistance in asthma^25^, doubled the median time of cardiac allograft survival^26^, and mediated tolerance to transplanted pancreas islet cells even without immune suppression^27^. A clear assessment of MDSC function, however, is complicated by the heterogenous nature of these cells, comprised of granulocytic and monocytic subtypes^26^.

As mice are typically immune-stimulated and sacrificed to generate MDSC, it is unclear whether sufficient cell numbers can even be isolated from living human donors. Human in vivo MDSC priming would also be severely limited by not only the ethical and clinical implications of inflammatory priming of organ donors, but possibly also by the freeze-thaw sensitivity of MDSC^28^.

**Fig. 2:**
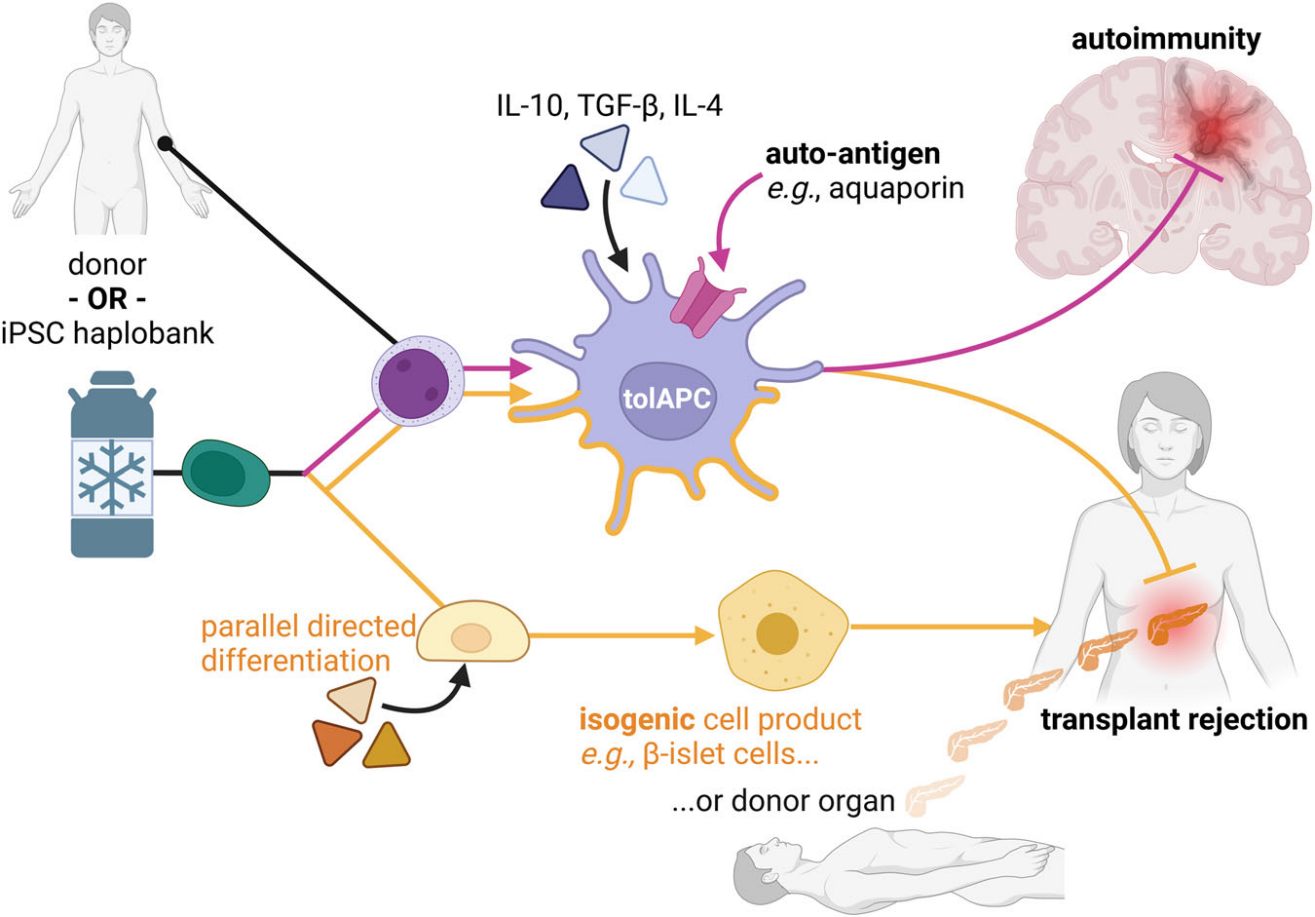
Engineered myeloid-derived antigen-presenting cells for the induction of tolerance to isogenic/self, autoreactive, or donor antigens. Myeloid APC derived from a cell donor or differentiated from hiPSC could be primed towards tolerance induction and loaded with autoreactive antigens to reduce autoimmunity (magenta). When organ and APC are derived from the same donor or differentiated from the same HLA-haplotyped hiPSC line, the APC could confer self-tolerance to the transplanted or other cell therapy product (yellow).

More specific and better-defined antigen presenting cells (APCs) might be preferred for therapy applications^23^. In liver and kidney transplantation^4,29^, multiple early clinical studies have demonstrated the feasibility and safety of autologous and allogenic APC for tolerance induction. In comparison to standard regimen, these promise less viral infections despite a reduced need for traditional immunosuppressives^4^. In autoimmunity, Zubizarreta et al provided proof-of-principle for the human in vivo application of tolerogenic DC differentiated from autologous monocytes against neuromyelitis optica after tolerogenic priming (IL-4) and loading with target autoantigens^3^ (Fig. 2, magenta arrows). This platform can probably be expanded to many other diseases with a known autoantigen or small group of autoantigens, such as autoimmune hemophilia or myasthenia gravis.

Instead of tedious autologous cell production and one-time loading with the antigen, hiPSC could be gene-edited to stably express the target antigen for an extended duration of effect^30^. Furthermore, hiPSC-derived DC or macrophages could convey “self-tolerance” to HLA-haploidentical transplant-cells, such as hiPSC-derived cardiac tissue, retina, or pancreatic islet cells, derived from the same hiPSC line (Fig. 2, yellow arrows). By relying on HLA-homozygous haplobanks, such a strategy combined with post-transplant cyclophosphamide (PtCy) protocols for haploidentical BMT^31^, may strongly expand the HLA-compatibility of hiPSC products. Indeed, Taylor et al have estimated, that as few as ten selected HLA-homozygous donors could provide a beneficial HLA-match to the majority of the UK population^32^.

## CAR-myeloid cells and pro-inflammatory cell priming

Over the last decade, CAR T-cells have gained significant traction in hematology-oncology. By expressing a fusion-construct of a highly avid binding site, elements of the T-cell receptor, and its obligate co-factors CD3 and CD28, these bioengineered cells circumvent the need for a double activation signal that prevents natural T-cells from overacting against self-antigens, and weaponizes this overreaction against cells carrying the target antigen, like B-cell lymphoma carrying CD19^33^. Upon encountering their target antigen, CAR-T-cells attack the target cells and clonally amplify in the presence of target antigens. However, tumor-escape by silencing the target antigen, and an immunosuppressive microenvironment of hypoxia and anti-inflammatory tumor-associated myeloid cells present obstacles, especially in solid tumors^33^.

A possible solution might be the parallel or sole application of CAR-myeloid cells. Although T-cell-specific CD3/CD4-receptors are not canonically involved in the activation of neutrophils and macrophages, the intracellular domain of CD3 shares sequences with Fc-receptors^34^, and their downstream signaling pathway converges with multiple innate myeloid immune signaling pathways via NFκB-activation; thus allowing for these unorthodox chimeras. Unlike T-cells, neutrophils and macrophages are not dependent on dual activation signaling and are not capable of extensive proliferation following activation. However, CAR-myeloid cells may not only attack and phagocytose tumor cells, but also intensely modulate the lymphocytic anti-tumor response^35,36^.

**Fig. 3:**
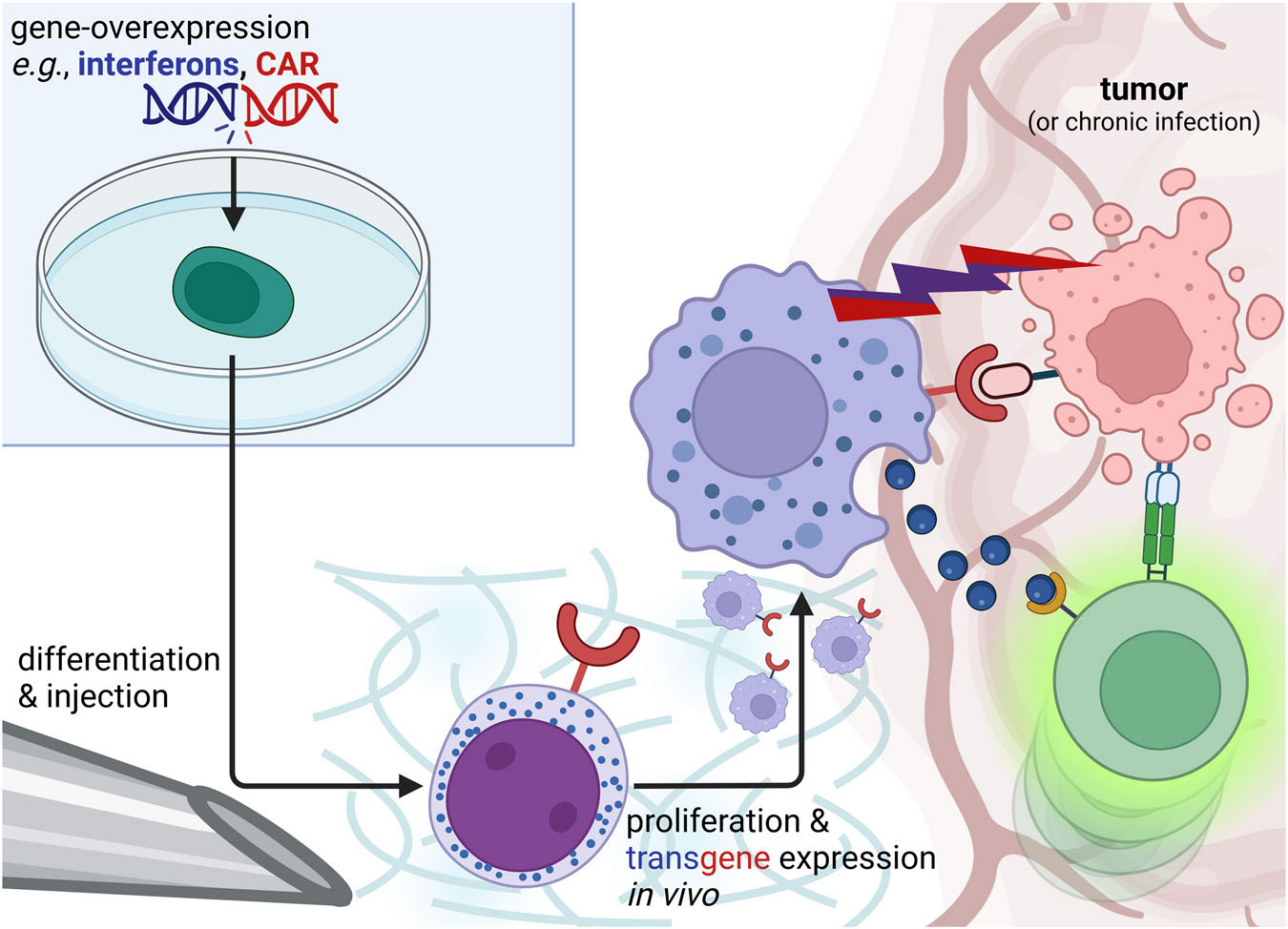
Gene-edited myeloid cells for the induction of comprehensive anti-tumor or anti-infectious immune responses. The microenvironment of solid tumors can suppress T-cell tumor immunity. Receptors against tumor antigens (e.g., CAR; red), or anti-tumor and immune stimulatory biodrugs (blue) can be stably expressed in hiPSC before myeloid differentiation. After injection and engraftment into an artificial or natural (e.g., bone-marrow, spleen, tumor tissue) scaffold, cells proliferate and home to the tumor (or a site of infection) to attack tumor cells directly and prime endogenous T-cells to overcome the anti-inflammatory microenvironment.

In a murine glioblastoma model, CD3/CD4/chlorotoxin second generation CAR-neutrophils exhibited greater tumor lysis and a 25%-longer host-survival compared to treatment with similarly devised CAR-NK-cells^37^. Similarly, hiPSC-derived CAR-macrophages improved CD8 T-cell amplification and chemotaxis, and reduced the tumor-burden in multiple murine ovarian cancer models; even achieving lasting responses under some conditions^34,36^ (Fig. 3, red cue); although farther exploration of how CAR-mediated myeloid cell activation shapes adaptive tumor immunity will be necessary, since most current studies relied on co-cultured in vitro systems or murine mutants without functional lymphoid cells.

Insufficient therapy persistence was a limiting factor in several CAR-neutrophil studies and required biweekly infusions^5^. While macrophages and related cell types can survive significantly longer than neutrophils, their long-term persistence is still limited^36^. A longer duration of effect can potentially be achieved by transfusing CAR-myeloid *progenitors* instead of differentiated effector cells^38^. Furthermore, granulocyte-macrophage progenitors can produce matched CAR-neutrophils and macrophages^39^, which could potentiate the effect on the tumor microenvironment or on bacterial killing. It may even be possible to steer the numbers of macrophages or granulocytes produced by administering appropriate growth factors (e.g., GCSF vs. granulocyte-macrophage stimulating factor vs. macrophage-colony stimulating factor), although, in an oncology setting, this is not without risk^40^.

The introduction of proliferation- and differentiation-inducing CARs^41^ may furthermore allow for self-regulating systems that produce effector cells in vivo; for only as long as a certain target antigen is present^41^. Potential risks of therapy persistence or malignant transformation could be mitigated by transfecting proliferation-inducing CAR-myeloid cells via non-integrating mRNA or by introducing ‘safety switches’ to improve product safety^42^. For example, by tying CAR-induced proliferation to the presence of a specific pharmaceutical substance, expansion of CAR-myeloid cells could be made contingent on the continued administration of a pharmaceutical “dead-man” switch.

To further expand the long-term in vivo production of cell therapies, injectable or implantable scaffolds could provide an optimal protective environment^43^ for the amplification, proliferation, and release of effector cells. Moreover, such bioengineering could augment attraction of host immune cells to enhance cell-cell communication, persistence, and effectiveness. For example, such scaffolds have previously been preloaded with extrinsic DC and CAR T-cells for in vivo amplification^44,45^. As the lifespan of certain populations (e.g., DC) may be limited, loading with longer-lived myeloid progenitors in situ (e.g., tumor, infection sites) may augment tumor vaccine approaches.

To increase immunity against a tumor independent of CAR, myeloid cells could be blinded against defense mechanisms, for example by blocking the CD47-SIRPα axis^46^. Alternatively, adaptive immunity could be enhanced to a tumor antigenic target via introduction of pro-inflammatory DC. For example, transfusion of nanoparticles (instead of whole cells) was employed to safely administer fusion-membranes of proinflammatory DC and tumor cells, to produce a personalized tumor vaccine^47^. While a direct translation of this method may be hindered by safety concerns, myeloid progenitors could be engineered to differentiate to proinflammatory DC that constitutively express target antigens to prime host T-cells against the malignancy.

Beyond tumor therapy, a short-term, limited boost of targeted CAR-neutrophils or CAR-macrophages may be useful for treating chronic infections (e.g., mycobacteriosis or multi-drug resistant bacterial infections). As both neutrophils and macrophages are simultaneously capable of promoting either mycobacterial killing or growth^48,49^, effective CAR-engineering may allow tipping this balance towards an anti-mycobacterial effect. Additionally, cells could be engineered to phagocytose and kill bacteria more effectively, or to prevent intracellular reproduction and persistence.

## Cellular biofactories for targeted drug delivery

Due to their effective homing capacities beyond physiological barriers^5,50^, neutrophils are uniquely well-suited to deliver a range of bioengineered substances to sites of inflammation^10^. A neutrophil-based delivery system^10,50,51^ offers three key advantages: reaching sites, that may otherwise not be amenable to a pharmaceutical^50^, enriching the substance on site beyond what’s systemically achievable^5^, and a double-barreled attack of cell therapy and pharmaceutical against tumors or infectious agents. However, neutrophil drug-carriers suffer from short lifespans and cannot be frozen, thus complicating logistics, limiting their duration of effect, and requiring frequent re-transfusions^5^. Instead, for many applications, mere coating of drug-carrying nanoparticles with myeloid cell membranes enabled effective delivery to sites of inflammation^52^. Coating of a promiscuous carrier, like polylactic-co-glycolic acid, allowed for a wide range of payloads including nucleic acid drugs, radionuclides, and petrochemically produced small molecules. Not relying on viable cells in the finished therapy product simplifies manufacturing, quality control, and logistics, and circumnavigates safety concerns of infusing viable cells, especially as particle-sizes typically allow for sterile filtration. However, this also eliminates the advantages of viable drug carriers, namely active movement beyond barriers and through tissue, and potentially amplification and drug-production on site.

Accordingly, instead of drug-loading cells or nanoparticles ex vivo, Wu et al engineered macrophages to produce IFN-γ in situ^53^. Similarly, transfused biofactories producing interferons, IL-12, and Tumor-Necrosis Factor were employed against melanoma, breast, cervical, and ovarian cancer, glioblastoma, and hematological malignancies in animal models^6,7,54^ (Fig. 3, blue cue), and are being explored against various tumor entities in early phase clinical studies^2,55^. A possible fear of a “cytokine storm” can be mitigated by triggering the mediator production or release on site or by tying drug production to specific promoters and thereby to specific macrophage subpopulations, like tumor-associated macrophages^2^. An advantage of this myeloid-based stimulation of the adaptive immune system over lymphoid-based approaches (e.g., CAR-T-cells) is the formation of endogenous tumor specific effector and memory T-cells, which may extend the effect far beyond persistence of the myeloid therapy product^6^ and make T-cell escape by surface antigen-change less likely.

Beyond signaling molecules, cells have been engineered to produce bispecific T-cell engagers or cytochrome P450 (toxic metabolites) and support tumor killing^56,57^. This approach of an endogenous biodrug production could be generalized to produce protein antibiotics^58^, anti-inflammatory^59^ or fatty acid signaling molecules, and nucleic acids, such as miRNA or aptamers^60^. To further expand on this concept, engineered myeloid immune cells can release pro- or anti-inflammatory extracellular vesicles, which have been demonstrated to reduce bacterial load and to improve survival in a murine colon ligature and puncture sepsis model^61^.

In summary, transfused, amplifying myeloid biofactories are a potentially promising platform for drug-delivery that may be suitable to many, albeit not all substances; polymer nanoparticles carrying radionuclides, and chemically produced small-molecules will likely continue to rely on ex vivo loading.

## Myeloid cell sourcing

**Fig. 4:**
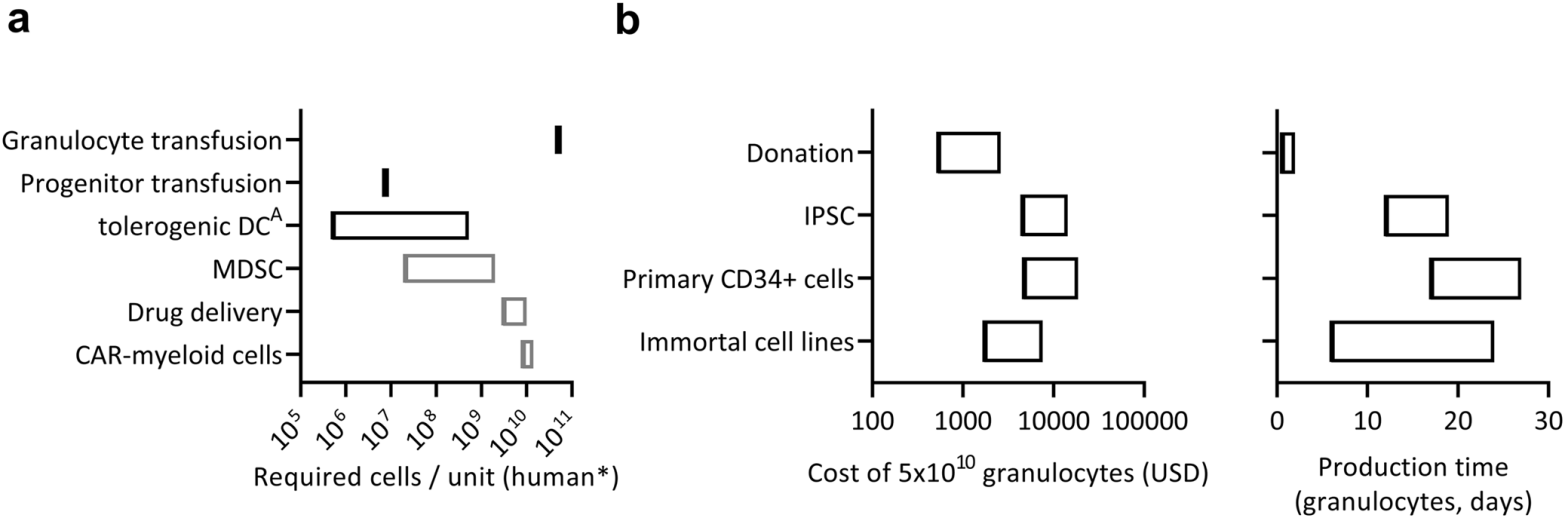
Versatility, economic, and logistical considerations for different applications and manufacturing approaches of myeloid therapies. **a** Estimated cell numbers required per therapeutic unit for different myeloid cell-based therapies. *Murine data was scaled by a factor of 2000; assuming 35 gm murine bodyweight and a human of 70 kg. Human (black) or murine (gray) in vivo data. DC, dendritic cells^**A**^, Includes studies with subcutaneous and intraperitoneal, instead of intravenous administration. **b** Delivery time and cost of 5 × 10^5^ neutrophils produced in cGMP-compliant conditions, excluding cost of irradiation (not applicable for progenitors), transport and product administration (highly variable). Details of data and methods for cost estimates are available in Supplementary Data 1.

Traditional methods to generate myeloid cells (e.g., cytokine-primed apheresis donation and buffy coat pooling) can be performed at every moderately-sized blood bank, although with variable quality and quantities between production sites^18^. Although adequate end-differentiated cells for tolerance-induction applications could likely be provided, these methods struggle to provide sufficient numbers of cells for treating neutropenia or for drug delivery applications (Fig. 4a; Supplementary Data 1). The resultant cell products are also difficult to standardize, are transport and storage sensitive, poorly amenable to cell engineering methods, limited in their duration of effect, and in the case of pooled donations, present high antigenic variability, and an increased risk of blood-borne infections.

In contrast, myeloid progenitors differentiated and amplified from hiPSC or from primary CD34^+^ cells^19,22,39^ can be gene-edited to express CAR, immune mediators, or self-/neoantigens, and can be frozen in large batches to simplify quality control and logistics. Moreover, myeloid progenitors can temporarily engraft and amplify, thus lowering the required cell numbers for extended therapy persistence.

Finally, hiPSC can produce any proposed cell therapy product^5,22,62,63^. Notably, the facile and stable genetic manipulation of hiPSC lines prior to directed myeloid differentiation via gene editing or transgene expression make them the most versatile vehicle for gene-modified products (e.g., introducing CAR-myeloid progenitors). Due to their almost unlimited self-renewal capacity, hiPSC provide a flexible and scalable^9^ cell source for myeloid therapies.

Unfortunately, derivation and validation of cGMP-grade hiPSC pose a considerable financial and regulatory hurdle. However, this caveat could be overcome by sourcing from banks of HLA-homozygous hiPSC derived in a cGMP-compliant manner^64^. Interline variability, an important issue when considering the feasibility of efficiently generating therapy products from a bank of hiPSC lines, may be overcome by employing more versatile hiPSC that eliminate lineage priming (e.g., tankyrase-inhibitor regulated naïve (TIRN) hiPSC lines)^65,66^; which may reduce the associated costs of protocol optimization and validation.

Another consideration prior to large-scale clinical implementation is product safety. Human hiPSC can acquire genetic and karyotypic mutations over extended culture^67^, even if these do not necessarily exceed natural genomic variability^68^. For engrafting hiPSC-derived hematopoietic cells, this has been linked to an increased propensity for malignant transformation^22^. Accordingly, functional research is often carried out in early passage hiPSC. Similar caution would be expected for hiPSC-manufactured therapy products. However, even when limiting culture to ≤15 passages, a 20-fold expansion per passage of one hiPSC can give rise to about 10^19^ cells, or over 650 million therapy units of 5 × 10^10^ cells without even considering additional amplification during differentiation. Hence for practical purposes, hiPSC constitute an unlimited source of therapeutic cells. Additionally, the limited number of successful hiPSC clinical trials to date have thus far demonstrated a favorable safety profile without malignant transformation^67^. Modern hiPSC reprogramming uses non-integrating techniques (e.g., episomal, Sendai-virus, or mRNA-based) that circumvent the risks of carry-over oncogenes like *c-Myc* or *KLF-4*. As granulocyte-macrophage progenitors and common lymphoid progenitors have limited self-renewal capacity and only engraft for weeks-to-months, they carry a very low risk of malignant transformation. Even in the context of BMT, product purity without contamination of long-term engrafting competent progenitors can be achieved by differential cultivation and phenotypic cell sorting^69^.

Primary CD34^+^ cells can be sourced from cord-blood, apheresis donation after mobilization, or from donor bone-marrow, and have been differentiated towards myeloid progenitors on a clinical scale^19,70^. While primary CD34^+^ cells can be genetically modified^39^, this approach is limited by their poor self-renewal and spontaneous terminal differentiation in vitro. While not requiring an additional initial investment to establish cGMP cell lines, repeated acquisition of primary CD34^+^ cells, for example in the form of cord-blood, accrues considerable running cost^8^; not even considering repeated validation of consecutive batches from different CD34+ seeds (Fig. 4b; Supplementary Data 1).

It will be interesting to evaluate whether hiPSC vs primary CD34^+^ cells will present the most affordable, versatile, and reliable option to generate the greatest numbers of therapeutic myeloid cells.

An alternative sourcing option may be the use of conditionally immortalized cell lines^39^. The leukemia cell line HL60 had historically been considered a possible cell source for granulocyte transfusions, due to its undemanding culture conditions suitable to bioreactor production and effective differentiation to granulocyte-like cells that share many functions with granulocytes^71,72^. While this cell line is not currently considered as a viable source of cells for transfusion, it may well serve as an inexhaustible cell source for applications in which viable cells can be clinically separated from patients (e.g., producing membrane-coated nanoparticles or ex vivo phagocytes in sepsis^72^). Beyond that, conditional immortalization of hiPSC-derived myeloid progenitors^39^ for unlimited bioreactor production of terminally differentiated cells without impaired functionality may be possible but will likely trigger safety concerns.

## Open research questions and future challenges

Several open questions remain before a large-scale therapeutic implementation of engineered myeloid progenitors becomes feasible. Importantly, while clinical data for neutrophil replacement is promising, an important question from animal experiments is whether myeloid progenitors can effectively increase the concentration of effector cells in the peripheral blood circulation^39^ or alternatively preferentially engraft and isolate to the spleen^73^. As the Desai et al studies did not provide peripheral blood granulocyte concentrations or biopsy data to elucidate these possibilities^19,70^, this question remains unresolved until future clinical studies. In addition, the immunological priming of transfused progenitors in humans warrants further investigation, as in mice, neutrophil progenitors largely differentiated to immune modulatory myeloid cells, which lessened septic inflammation^74^.

Another open question is: when and for how long is the optimal time to transfuse neutrophil, granulocyte-macrophage, or common myeloid progenitors? Some applications, like bridging neutropenia, may only rely on transient neutrophil transfusion, while manipulating the tumor microenvironment may rely on persistence of longer-lived myeloid cells. This question is contrasted further by the differential lifespan and required cell numbers of different myeloid cell types and applications. While the production and transfusion of terminally differentiated neutrophils is barely feasible clinically, macrophages can potentially survive in culture long enough for gene editing and cryopreservation. Since macrophages can also exert an in vivo effect over a few weeks^7,36^, progenitor transfusion may not be necessary for macrophage-based therapies. Nonetheless, it may still be advantageous to use hiPSC-derived myeloid progenitors to simplify macrophage engineering, and to replace expensive autologous therapies with “off-the-shelf” hiPSC-based cell bank approaches. Accordingly, future studies are required to answer the question of which cell-type, end-differentiated macrophage or DC, or a myeloid progenitor is best suited for specific therapy applications.

Although recent studies suggest a negligible role of HLA-matching^19,21^ for neutrophil and progenitor transfusions, the specific time to clearance of engineered myeloid progenitors will likely ultimately depend on HLA-matching, serological cross-reactivity, and immune competency or graft recovery in the case of BMT. For macrophages, the situation is even less clear, as most studies employed an autologous approach or did not provide a side-by-side comparison^1,3,4,75^. Although both hiPSC and primary CD34^+^ cells could be collected for autologous therapy, decreased batch size, increased validation costs, and time of manufacturing would limit their economic feasibility^8,76^. When considering engineered myeloid progenitors, the presumptive safety benefits of HLA-mismatched transfusions must hence be weighed against an expected shorter duration of in vivo effect. Many strategies have been developed to avoid immune detection or attack by the host and thus extend cell therapy persistence. These methods, subsumed under the term “hypo-immune cell engineering”, usually rely on the knockout of multiple HLA-alleles, often combined with overexpression of immune-regulatory signaling receptors, like the ”don’t-eat-me” receptor CD47, or of regulatory HLA-subtypes E or G^77^. Although longevity of transfused myeloid progenitors could be modified by HLA- or other hypo-immune engineering, many of the myeloid cell functions discussed here rely on their antigen-presentation capacities via HLA. Thus, hypo-immune engineering of cell therapies may only be appropriate for a limited range of applications, and the use of HLA-homozygous hiPSC cell banks might ultimately be more broadly feasible^32^. Important remaining research questions include, which applications are majorly affected by non-HLA matching, and whether HLA-haplobanking or hypo-immune engineering can circumvent these challenges.

Myeloid cell therapies hold immense promise in hematology-oncology, immunology, and infectious disease disciplines with many novel uses emerging beyond mere cell replacement (Figs. 2, 3). Granulocytes and macrophages can be modified to support tumor-killing through the introduction of CAR. Due to their effective homing to inflamed tissues, myeloid cell-based carriers could also be exploited to produce and deliver anti-tumor or anti-microbial bio-drugs and achieve greater drug concentrations in diseased niche sites. Cells carrying healing signals, immature subtypes like MDSC, or tolerogenic macrophages or DC could be exploited to dampen compartment-specific inflammation in sepsis, asthma, arthritis, autoimmunity, or allogeneic transplant rejection, thus obviating the need for systemic immune suppression. However, donation-derived granulocytes and macrophages cannot be produced in sufficient cell numbers for many of these applications, and their short lifespan further complicates logistics and efficacy. Alternatively, multipotent myeloid progenitors manufactured from hiPSC or from primary CD34^+^ may not only alleviate these sourcing difficulties, but also extend the duration of effect, greatly simplify cell engineering, and allow an “off-the-shelf” provision, due to superior in vivo expansion capacities and improved cryo-preservation tolerance. Thus, bio-engineered, short-term engrafting myeloid progenitors could reduce required cell numbers and help bring bioreactor volumes down to a practical scale for making their production more affordable. We propose that these collective advantages ultimately outweigh the higher initial investment for establishing engineered hiPSC lines or harvesting donor primary CD34^+^ PBSC.

### Supplementary information

Supplementary information

## Data Availability

All data analyzed in this study are included in this published article and its Supplementary Information files.
