# Supplementary material for: Harnessing bioengineered myeloid progenitors for precision immunotherapies
Source: NPJ Regen Med. 2023 Dec 12;8:66. doi: 10.1038/s41536-023-00343-x (PMC10716389; doi:10.1038/s41536-023-00343-x)
Supplement: Supplementary file 1 — Supplementary information [file 41536_2023_343_MOESM1_ESM.pdf]

Supplementary Information:

Harnessing Bioengineered Myeloid Progenitors for Precision Immunotherapies

Willem Buys and Elias T. Zambidis

A. Supplementary Data

Supplementary Data 1: Comparative Methods and Cost Estimates for Various Myeloid Bioengineering Approaches.

a. Neutropenia incidence estimates (see **Table 1**)

Absolute numbers are based on the US-population and birth-rate according to the United States Census Bureau [1]. Data on the U.S. incidence of cancer, cancer death, and bone-marrow transplantation is derived from the U.S. Center for International Blood And Marrow Transplantation Research and the National Cancer Institute [2,3]. Incidence data for associated severe neutropenia is derived from [4,5]. The incidence of non-chemotherapy induced neutropenia and of neonatal sepsis is derived from [6,7].

b. Required cell numbers per therapy modality (see **Figure 4a**)

Data derived from [8-16].

c. Delivery time and cost estimates (see **Figure 4b**)

The production cost and time of terminally differentiated neutrophils is based on a high vs. low cost and time scenario as proxy due to the lack of high-quality clinical scale data for progenitor production. Delivery time is defined as production time on demand, or time between the last timepoint, at which the cells can be cryopreserved, to the earliest possible delivery.

The running costs for cryostorage of hiPSC seeding batches is negligible [17] compared to the acquisition of $CD34^+$ cells [18] and hiPSC derivation [19]. All calculations assume a conservative material cost reduction of 30% for bulk ordering [18].

The tables below present values rounded to the next whole numbers. Calculations were performed with the exact values. Prices as published or market prices as of September 2023.

d. Primary $CD34^+$ cells; high-cost estimate:

Delivery time and cost of $CD34^+$ derived myeloid cells according to [18], the main variable being the cost and quality of the available primary $CD34^+$ cells.

High material-cost estimate	7448 USD
- 30% discount for bulk ordering	5214 USD
+ 43% non-material cost at higher cord-blood price [18]	7477 USD
Adjustment factor for cell number x 2.5	18690 USD
**Total cost**	**18690 USD**

e. Primary $CD34^+$ cells; low-cost estimate:

Low material-cost estimate, includes bulk ordering discount	1607 USD
+ 16% non-material cost at lower cord-blood price	1876 USD
Adjustment factor for cell number x 2.5	4690 USD
**Total cost**	**4690 USD**

f. Donation; high-cost estimate:

The cost of donated concentrates is estimated as cost of apheresis-donated platelets (procedural cost) plus cost per dose of G-CSF [20,21]. Cell numbers [9,22,23].

Procedural cost of apheresis [20] [A]	730 USD
G-CSF [21] [A,B]	490 USD
Pre-visit for G-CSF administration	80 USD
Preliminary sum	1300
Adjustment factor for insufficient cell numbers [9,22]	x 2
**Sum**	**2600**

g. Donation; low-cost estimate:

Procedural cost of apheresis [20]	492 USD
G-CSF biosimilar [21] [B]	102 USD
Self-administration	0 USD
**Sum** [C]	**530**

h. Donation costs; published data:

Apheresis: 1709 USD [24] [A,B,C]

Pooling:

Single granulocyte fraction [25]; 9x10^8 cells [23]	40 USD
Adjustment factor for cell numbers	x55
**Total cost**	**2200**

[A] Cost adjusted for inflation in USD since publication to 09/23 using [26].

[B] Calculated from a published cost in another currency by the exchange rate at the time of publication using [27].

[C] assuming sufficient cell numbers [9].

i. Immortal cell lines; high-cost estimate:

The cost of HL60 myeloid cells is estimated following [28,29]: Cell density at harvest $2x10^6$ cells/ml X-VIVO 15 (high-cost scenario) or Iscove's Modified Dulbecco's Medium (IMDM, low-cost scenario) with 1 µmol/l trans-retinoic acid in a 50-liter bioreactor. Doubling time 2-3 days without cell loss during differentiation [28]. Five days of differentiation plus recovery day after thawing (short scenario) or including HL60 amplification from a seed of 10^8 cells (long scenario).

At a harvesting density of $2x10^6$ HL60 cells per ml, a single 50 L bioreactor could produce two therapy units of $5x10^{10}$ cells per batch.

50 L X-Vivo 15 medium at 400 USD / L	20,000 USD
Trans-retinoic acid priced at 334 USD / 5 gm.<br>Target concentration 1 µmol/l in 50 L.	20,001 USD
Adjustment factor for cell number x 0.5	10,001 USD
- 30% bulk-ordering discount	7001 USD
+9% non-material cost	7609 USD
**Total cost**	**7609 USD**

j. Immortal cell lines; low-cost estimate:

50 L IMDM at 90 USD / L	4500 USD
Trans-retinoic acid priced at 334 USD / 5 gm.<br>Target concentration 1 µmol/l in 50 L.	4501 USD
Adjustment factor for cell number x 0.5	2251 USD
- 30% bulk ordering discount	1576 USD
+9% non-material cost	1712 USD
**Total cost**	**1712 USD**

k. hiPSC culture materials and supplies:

There is no published clinical scale protocol for neutrophil production from hiPSC. However, a scalable bioreactor protocol for the production of macrophages has been adapted to neutrophils on a smaller scale [30-32]. Torres-Acosta, et al [18] calculated the cost of neutrophil production from primary $CD34^+$ cells based on the Timmins, et al. protocol [33]; also see primary CD34-cells above. Adjusted for the production per time, this protocol produces ca. 2-fold less cells per batch but 2-fold more cells per culture volume, than the Lachmann, Ackermann, et al. protocol. It uses a 25% more expensive base medium and two / one more growth factor in the initial / main phase of cell production, respectively. Additionally, Torres-Acosta, et al. found material costs to be the main driver of costs in 5 out of 6 scenarios of neutrophil production from primary $CD34^+$ cells.

Comparison of drivers of material cost between production from hiPSC vs primary CD34 cells:

	Torres-Acosta, et al. [18]	Lachmann, Ackermann, et al. [30-32]
Main medium, price*	Stemline 2, 500 USD / L	X-Vivo 15, 400 USD / L
Growth-factors, initial	SCF, TPO, FLTK3L	bFGF
Growth-factors, main	SCF, TPO_{pep}, G-CSF	IL-3, G-CSF
Bioreactor volume	104 L	40 – 60 L
Cell number / days	$20x10^{10}$ cells / 27 days	10^{10} cells / 7 days

* listed price 09/2023

It can thus be assumed, that the cost of goods for the production of neutrophils from hiPSC per cell number is similar to the production from primary $CD34^+$ cells. Human iPSC accrue no running cost for the repeated acquisition of cord-blood cells. However, establishing a cGMP line costs about 800,000 USD [19]. This adds about 114 USD per therapy unit of $5x10^{10}$ cells when offset over one year of cell production at ≈10% market penetration in the USA (7000 units, also see **Table 1**)

l. hiPSC; high-cost estimate:

High material-cost estimate by [18]	7448 USD
-30% bulk ordering discount	5214 USD
+ 9% non-material cost (no cord-blood)	5667 USD
Adjustment factor for cell number x 2.5	14167 USD
Offsetting establishing the hiPSC line +114 USD	14281 USD
**Total cost**	**14281 USD**

m. hiPSC; low-cost estimate:

Low material-cost estimate by [18]	1607 USD
Includes bulk ordering discount	1607 USD
+ 9% non-material cost (no cord-blood)	1747 USD
Adjustment factor for cell number x 2.5	4367 USD
Offsetting establishing the hiPSC line +114 USD	4481 USD
**Total cost**	**4481 USD**

Delivery time of hiPSC-derived neutrophil estimated on the basis of [18,30-32].

B. Supplementary References

1. Bureau USC (2023) U.S. and World Population Clock. United States Census Bureau. https://www.census.gov/popclock/. Accessed 18.05.2023 2023

2. Transplant CfIBaM (2022) Transplant Activity Report. vol 2023. for the C.W. Bill Young Cell Transplantation Program,

3. NCI (2020) Cancer Statistics vol 2023. National Cancer Institute, National Institutes of Health (NIH), US Department of Health and Human Services (HHS),

4. Jolis L, Carabantes F, Pernas S, Cantos B, López A, Torres P, Funes C, Caballero D, Benedit P, Salar A (2013) Incidence of chemotherapy-induced neutropenia and current practice of prophylaxis with granulocyte colony-stimulating factors in cancer patients in Spain: a prospective, observational study. Eur J Cancer Care (Engl) 22:513-521. doi:10.1111/ecc.12057

5. Weycker D, Barron R, Kartashov A, Legg J, Lyman GH (2014) Incidence, treatment, and consequences of chemotherapy-induced febrile neutropenia in the inpatient and outpatient settings. J Oncol Pharm Pract 20:190-198. doi:10.1177/1078155213492450

6. Curtis BR (2017) Non–chemotherapy drug–induced neutropenia: key points to manage the challenges. Hematology 2017:187-193. doi:10.1182/asheducation-2017.1.187

7. Celik IH, Hanna M, Canpolat FE, Mohan P (2022) Diagnosis of neonatal sepsis: the past, present and future. Pediatric Research 91:337-350. doi:10.1038/s41390-021-01696-z

8. Brempelis KJ, Cowan CM, Kreuser SA, Labadie KP, Prieskorn BM, Lieberman NAP, Ene CI, Moyes KW, Chinn H, DeGolier KR, Matsumoto LR, Daniel SK, Yokoyama JK, Davis AD, Hoglund VJ, Smythe KS, Balcaitis SD, Jensen MC, Ellenbogen RG, Campbell JS, Pierce RH, Holland EC, Pillarisetty VG, Crane CA (2020) Genetically engineered macrophages persist in solid tumors and locally deliver therapeutic proteins to activate immune responses. J Immunother Cancer 8. doi:10.1136/jitc-2020-001356

9. Price TH (2014) The RING study: A Randomized Controlled Trial of GCSF-stimulated Granulocytes in Granulocytopenic Patients. Blood 124:SCI-16-SCI-16. doi:10.1182/blood.v124.21.sci-16.sci-16

10. Ravandi F, Abboud CN, Akard LP, Gill SI, Hsu JW, Kambhampati S, Khan I, Liu D, Stock W, Brown JW, Bashey A, Borthakur G, DiNardo CD, Holland HK, Kadia TM, Kantarjian HM, Morris LE, Solomon SR, Mamelok RD, Panuganti S, Van Syoc R, Wong A, Zimmerman D, Solh M (2018) Evaluation of Romyelocel-L Myeloid Progenitor Cells to Decrease Infections in De Novo AML Patients Receiving High-Dose Ara-C-Based Induction Therapy. Blood 132:1407-1407. doi:10.1182/blood-2018-99-117231

11. Morales-Mantilla DE, Kain B, Le D, Flores AR, Paust S, King KY (2022) Hematopoietic stem and progenitor cells improve survival from sepsis by boosting immunomodulatory cells. Elife 11. doi:10.7554/eLife.74561

12. Suuring M, Moreau A (2021) Regulatory Macrophages and Tolerogenic Dendritic Cells in Myeloid Regulatory Cell-Based Therapies. Int J Mol Sci 22. doi:10.3390/ijms22157970

13. Cai S, Choi JY, Borges TJ, Zhang H, Miao J, Ichimura T, Li X, Xu S, Chu P, Eskandari SK, Allos H, Alhaddad JB, Muhsin SA, Yatim K, Riella LV, Sage PT, Chandraker AK, Azzi JR (2020) Donor myeloid derived suppressor cells (MDSCs) prolong allogeneic cardiac graft survival through programming of recipient myeloid cells in vivo. Sci Rep 10:14249. doi:10.1038/s41598-020-71289-z

14. Klichinsky M, Ruella M, Shestova O, Lu XM, Best A, Zeeman M, Schmierer M, Gabrusiewicz K, Anderson NR, Petty NE, Cummins KD, Shen F, Shan X, Veliz K, Blouch K, Yashiro-Ohtani Y, Kenderian SS, Kim MY, O'Connor RS, Wallace SR, Kozlowski MS, Marchione DM, Shestov M, Garcia BA, June CH, Gill S (2020) Human chimeric antigen receptor macrophages for cancer immunotherapy. Nat Biotechnol 38:947-953. doi:10.1038/s41587-020-0462-y

15. Chang Y, Syahirah R, Wang X, Jin G, Torregrosa-Allen S, Elzey BD, Hummel SN, Wang T, Li C, Lian X, Deng Q, Broxmeyer HE, Bao X (2022) Engineering chimeric antigen receptor neutrophils from human pluripotent stem cells for targeted cancer immunotherapy. Cell Rep 40:111128. doi:10.1016/j.celrep.2022.111128

16. Klinkmann G, Wild T, Heskamp B, Doss F, Doss S, Arseniev L, Aleksandrova K, Sauer M, Reuter DA, Mitzner S, Altrichter J (2022) Extracorporeal immune cell therapy of sepsis: ex vivo results. Intensive Care Med Exp 10:26. doi:10.1186/s40635-022-00453-8

17. Beth Sissons, Arquilla E (2020) Cord blood banking: Cost, pros, and cons. https://www.medicalnewstoday.com/articles/cord-blood-banking. Accessed 20.04.2023 2023

18. Torres-Acosta MA, Harrison RP, Csaszar E, Rito-Palomares M, Brunck MEG (2019) Ex vivo Manufactured Neutrophils for Treatment of Neutropenia-A Process Economic Evaluation. Front Med (Lausanne) 6:21. doi:10.3389/fmed.2019.00021

19. Bravery C (2015) Do Human Leukocyte Antigen-Typed Cellular Therapeutics Based on Induced Pluripotent Stem Cells Make Commercial Sense? Stem Cells and Development 24:1-10. doi:10.1089/scd.2014.0136

20. Barnett CL, Mladsi D, Vredenburg M, Aggarwal K (2018) Cost estimate of platelet transfusion in the United States for patients with chronic liver disease and associated

thrombocytopenia undergoing elective procedures. Journal of Medical Economics 21:827-834. doi:10.1080/13696998.2018.1490301

21. Hadji P, Kostev K, Schröder-Bernhardi D, Ziller V (2012) Cost comparison of outpatient treatment with granulocyte colony-stimulating factors (G-CSF) in Germany. International journal of clinical pharmacology and therapeutics 50:281-289. doi:10.5414/CP201633

22. Marfin AA, Price TH (2015) Granulocyte transfusion therapy. J Intensive Care Med 30:79-88. doi:10.1177/0885066613498045

23. Morton S, Fleming K, Stanworth SJ (2022) How are granulocytes for transfusion best used? The past, the present and the future. Br J Haematol. doi:10.1111/bjh.18445

24. Gustavsson E, Sjödahl R, Theodorsson E (2020) The ethical dilemma of granulocyte transfusions. Clinical Ethics 15:156-161. doi:10.1177/1477750920927162

25. Cost of blood components. Fairview. https://www.fairview.org/transfusion/cost-of-blood-components. Accessed 19.04.2023 2023

26. Inflation calculator. U.S. Bureau of labor statistics. https://www.bls.gov/data/inflation_calculator.htm.

27. Exchangerate.org. UK FX Ltd. https://www.exchangerates.org.uk/EUR-USD-spot-exchange-rates-history-2011.html.

28. Guo Y, Gao F, Wang Q, Wang K, Pan S, Pan Z, Xu S, Li L, Zhao D (2021) Differentiation of HL-60 cells in serum-free hematopoietic cell media enhances the production of neutrophil extracellular traps. Exp Ther Med 21:353. doi:10.3892/etm.2021.9784

29. Babatunde KA, Wang X, Hopke A, Lannes N, Mantel P-Y, Irimia D (2021) Chemotaxis and swarming in differentiated HL-60 neutrophil-like cells. Scientific Reports 11. doi:10.1038/s41598-020-78854-6

30. Ackermann M, Rafiei Hashtchin A, Manstein F, Carvalho Oliveira M, Kempf H, Zweigerdt R, Lachmann N (2022) Continuous human iPSC-macrophage mass production by suspension culture in stirred tank bioreactors. Nat Protoc 17:513-539. doi:10.1038/s41596-021-00654-7

31. Ackermann M, Kempf H, Hetzel M, Hesse C, Hashtchin AR, Brinkert K, Schott JW, Haake K, Kuhnel MP, Glage S, Figueiredo C, Jonigk D, Sewald K, Schambach A, Wronski S, Moritz T, Martin U, Zweigerdt R, Munder A, Lachmann N (2018) Bioreactor-based mass production of human iPSC-derived macrophages enables immunotherapies against bacterial airway infections. Nat Commun 9:5088. doi:10.1038/s41467-018-07570-7

32. Lachmann N, Ackermann M, Frenzel E, Liebhaber S, Brennig S, Happle C, Hoffmann D, Klimenkova O, Luttge D, Buchegger T, Kuhnel MP, Schambach A, Janciauskiene S, Figueiredo C, Hansen G, Skokowa J, Moritz T (2015) Large-scale hematopoietic differentiation of human induced pluripotent stem cells provides granulocytes or macrophages for cell replacement therapies. Stem Cell Reports 4:282-296. doi:10.1016/j.stemcr.2015.01.005

33. Timmins NE, Palfreyman E, Marturana F, Dietmair S, Luikenga S, Lopez G, Fung YL, Minchinton R, Nielsen LK (2009) Clinical scale ex vivo manufacture of neutrophils from hematopoietic progenitor cells. Biotechnol Bioeng 104:832-840. doi:10.1002/bit.22433
